# Overexpression of long noncoding RNA HOXB-AS3 indicates an unfavorable prognosis and promotes tumorigenesis in epithelial ovarian cancer via Wnt/β-catenin signaling pathway

**DOI:** 10.1042/BSR20190906

**Published:** 2019-08-02

**Authors:** Xiao-hong Zhuang, Ying liu, Jin-ling Li

**Affiliations:** 1 Department of Obstetrics and Gynecology, People’s Hospital of Linyi Economic Development Area, Linyi, Shandong, China; 2 Department of Gynecology, The Affiliated Qingdao Hiser Hospital of Qingdao University, Qingdao, Shandong, China; 3 Department of Obstetrics and Gynecology, Hedong People’s Hospital, Linyi, Shandong, China

**Keywords:** HOXB-AS3, Long noncoding RNA, Metastasis, Prognosis, Proliferation, Wnt/β-catenin signaling

## Abstract

Long noncoding RNA HOXB cluster antisense RNA 3 (HOXB-AS3) has been reported to be dysregulated in several tumors. The present study mainly aims at the investigation in how HOXB-AS3 works in epithelial ovarian cancer (EOC) and to elucidate the mechanism involved. Initially, ‘GEPIA’ was mined to examine the differential expression levels and prognostic value of HOXB-AS3 in EOC patients. The expression of HOXB-AS3 in EOC cell lines and patient specimens was examined with quantitative RT-PCR. Simultaneously, the correlation of HOXB-AS3 expression with a variety of clinicopathological factors and patient survival was analyzed. MTT, colony formation and flow cytometry assays were performed to analyze the cell viability of EOC cells. Wound healing and Transwell assays were carried out to determine EOC cells’ capability of migrating and invading. The impact of HOXB-AS3 on EMT and Wnt/β-catenin signaling was explored with the approach of Western blot. We found that in both EOC cell lines and tissues, HOXB-AS3 expression was significantly up-regulated, and its high expression was an independent prognostic marker of poor outcome for EOC patients. *In vitro* loss-of-function assays revealed that HOXB-AS3 knockdown inhibited EOC cells proliferation, migration, invasion and EMT, and induced EOC cells’ apoptosis. Furthermore, we validated that down-regulated HOXB-AS3 attenuated the activity of Wnt/β-catenin signaling to suppress the invasion, migration and proliferation of EOC cells. To sum up, the present study came up with the conclusion that HOXB-AS3 acts as an oncogenic gene in EOC progression through HOXB-AS3-Wnt/β-catenin signaling regulation, providing a novel insight into EOC tumorigenesis.

## Introduction

Human ovarian cancer, one of the most common gynecologic neoplasms, is responsible for the highest mortality among women, and represents a major concern for women’s health worldwide [1,2]. And nearly nine in ten female ovarian cancer patients are diagnosed with epithelial ovarian cancer (EOC) [3]. Despite the current EOC treatment approaches, such as radiotherapy, chemotherapy and operation, have considerably advanced and improved, the clinical outcome of EOC patients is still very unsatisfactory, which has reflected in a disappointing 5-year survival rate of only 38% among treated EOC patients [4–6]. The diagnoses of most EOC patients cannot be confirmed until the disease develops to a late stage, which is a major contributor to the unsatisfactory survival rate. Hence, great efforts need to be made to identify key molecules involved in the pathogenesis of EOC, which could help in EOC’s early detection and improvement of current treatment.

Long noncoding RNAs (lncRNAs) refers to a set of functional RNAs which generally consist of over 200 nucleotides without protein-encoding ability [7]. Growing evidences reveal that the dysregulated lncRNAs are associated with tumor progression and cellular process, including proliferation, apoptosis and cell differentiation [8,9]. It is reported that lncRNAs display their functions in a wide range of processes and can modulate the levels of genes by various mechanisms [10,11]. More importantly, a growing number of studies have indicated that the dysregulation of lncRNAs expression was involved in many cancers including EOC and served as tumor promoters or tumor suppressors according to the types of cancers [12,13]. Recently, several studies have explored the feasibility of using lncRNAs as diagnostic or prognostic biomarkers in tumors, including EOC [14–16]. However, the expression and function of most lncRNAs remain largely unknown.

LncRNA HOXB cluster antisense RNA 3 (HOXB-AS3) is a newly identified lncRNA whose dysregulation has been discovered and reported in a number of different cancers, including colon cancer, acute myeloid leukemia and lung adenocarcinoma [17–19]. However, its expression and function in cancers remains largely unclear. In the present study, we first performed an online tool to screen differently expressed lncRNAs and found that HOXB-AS3 was significantly highly expressed in EOC patients. Then, we further detected the expression and biological function of HOXB-AS3, as well as its prognostic value in EOC patients. Our findings provided novel insights into the molecular function of HOXB-AS3/Wnt/β–catenin signaling in EOC.

## Materials and methods

### Clinical specimens

EOC tissues and corresponding normal adjacent specimens were collected from EOC patients with EOC at The Second Affiliated Hospital of Hedong People’s Hospital from July 2009 to April 2013. The diagnosis and histological classification of samples were blindly determined by two independent pathologists. The histological diagnosis of EOC was evaluated according to the World Health Organization. Written informed consents were obtained from these patients. None of the patients received preoperative anti-cancer therapy. All clinical samples were stored using liquid nitrogen. The protocols were approved by the Ethics Committee of our hospital.

### Cell lines and reagents

IOSE80 (as control), SKOV-3, HEY and OVCAR-3 were bought from the Shanghai Cell Bank (Xuhui, Shanghai, China). DMEM was utilized to culture the cells (containing 10% FBS). In addition, 100 U/ml penicillin and 50 μg/ml streptomycin (Meilun, Haidian, Beijing, China) were also needed to add into the culture medium. Besides, Wnt/β-catenin signaling inhibitor (XAV-939) and agonist (SKL2001) were bought from Selleck Corporation (Pudong, Shanghai, China).

### Cell transfection

The siRNAs: si-NC (as control), si-HOXB-AS3#1 and si-HOXB-AS3#2, were bought from GenePharma Inc. (Xuhui, Shanghai, China). Invitrogen Lipofectamine 2000 kits (YingDi, Suzhou, Jiangsu, China) were used to conduct cell transfection. In short, SKOV-3 or OVCAR-3 cells (2 × 10^5^ cells/well) were cultured in 12-well plates to grow to approximately 60% confluence. Subsequently, 6 μl siRNA duplex (20 μM) was mixed with Lipofectamine 2000 regents (10 μl). After 20 min, the mixture was placed into the plates. The medium needed to be changed 5 h later.

### Quantitative RT-PCR assay

Total RNAs from EOC specimens and cells were isolated using TRIzol solution (AndeBio, Xuhui, Shanghai, China). Subsequently, 1 μg total RNA was reverse transcribed into cDNA using cDNA Synthesis kits (Transgen, Shijingshan, Beijing, China). Then, qPCR was conducted using a SYBR–Green Master Mix kit (TIANGEN, Haidian, Beijing, China) and performed by CFX96 qPCR machine (Invitrogen, Carlsbad, CA, U.S.A.). The housekeeping gene, *GAPDH*, was used as internal control. The primers are listed in Table 1. The 2^−ΔΔ*C*^_t_ method was used to calculate the relative fold changes.

**Table 1:** The primers for PCR

Primer name	Primer sequence (5′–3′)
β-catenin (F)	GTGTGGCGACATATGCAGC
β-catenin (R)	CAAGATCAGCAGTCTCATTC
cyclinD1 (F)	GTAGCAGCGAGCAGCAGAGT
cyclinD1 (R)	CGGTCGTGAGGAGGTTGG
c-myc (F)	TTCGGGTAGTGGAAAACCCAG
c-myc (R)	CAGCAGCTCGA ATTTCTTCC
HOXB-AS3 (F)	CCCTCCAAGTCCAGTAAGAAGT
HOXB-AS3 (R)	AGATCCTAAGAGGTGCGAGTTTA
GAPDH (F)	GGAGCGAGATCCCTCCAAAAT
GAPDH (R)	GGCTGTTGTCATACTTCTCATGG

### Western blot

Proteins were isolated using RIPA buffer (KaiGene, Nanjing, Jiangsu, China) and qualified by BCA kits (Solarbio, Haidian, Beijing, China). The proteins were then separated by dodecyl sulfate, sodium salt/polyacrylamide gel electrophoresis (SDS/PAGE) and transferred to polyvinylidene fluoride (PVDF) membranes. Target proteins were incubated with primary antibodies: Anti-Vimentin (#10366-1-AP, CST, Danvers, MA, U.S.A.), Anti-E-Cadherin (#sc-71008, CST, Danvers, MA, U.S.A.), Anti-N-Cadherin (#ab18203, Abcam, Pudong, Shanghai, China), Anti-Caspase-9 (#ab325539, Abcam, Pudong, Shanghai, China), Anti-Caspase-3 (#9662, CST, Danvers, MA, U.S.A.), Anti-β-catenin (#51067-2-AP, CST, Danvers, MA, U.S.A.), Anti-Cyclin D1 (#2922, Abcam, Pudong, Shanghai, China), Anti-c-Myc (#ab39688, Abcam, Pudong, Shanghai, China), Anti-GAPDH (#60004-1-Ig, CST, Danvers, MA, U.S.A.). Afterward, matched secondary antibodies was used to incubate the membranes, followed by examination using ECL analyses kits (KeenBio, Xuhui, Shanghai, China).

### Cell proliferation analyses

Cell proliferation was examined using MTT assay kits (Solarbio, Haidian, Beijing, China). In short, cells were placed in 96-well plates (1 × 10^3^ cells in each well). After attachment, MTT reagents (30 μl/well; 0.8 mg/ml) were added into the plates. After incubation for 3–4 h, 100 μl DMSO reagent (per well) was added into the two EOC cell lines. After incubating for 10 min, microplate reader was used to detect the absorbance at 490 nm.

### Clonogenic assay

Cells (500 cells/well) were placed in six–well plates and then incubated for a further 14 days. Then, 0.05% Crystal Violet (Solarbio, Haidian, Beijing, China) was applied to stain the cell colonies after being fixed with 4% paraformaldehyde. The colonies were counted using a camera (Nikon, Chiyoda-Ku, Tokyo, Japan).

### Flow cytometry analysis

For the cell apoptosis analyses, cells transfected with negative control siRNAs or HOXB-AS3 siRNAs were harvested at 48 h after transfection. Then, Annexin V- FITC (5 μl) and PI (5 μl) were used to double-stain the tumor cells in the dark for 15 min. Subsequently, FACS Calibur flow cytometry machine (BD Biosciences, Kunming, Yunnan, China) was utilized to analyze the number of apoptotic cells.

### Migration assay

Cell suspensions (60 μl, 8 × 10^5^ cells/ml) were added into each reservoir of the 35-mm high culture μ-dish (Ibidi, Planegg, Martinsried, Germany). After the cell attachment for 24 h, a gap of 500 μm was generated after removing the culture inserts using sterile tweezers. Then, 500 μl medium was added into each dish. The images of wounded areas were photographed at 0 and 48 h.

### Transwell assay

Transwell assay was conducted by the use of transwell plates with 8-μm pore inserts (Millipore, Shijingshan, Haidian, U.S.A.). Cells (250 μl; 3 × 10^5^ cells) were placed into the upper sides of the membranes (Matrigel-treated) without FBS, while the lower sides were added with medium plus 10% FBS. Twenty-four hours later, cells of EOC on the bottom sides of the membranes were fixed using 4% paraformaldehyde (Solarbio, Haidian, Beijing, China) and stained with 0.3% Crystal Violet dye (Solarbio, Haidian, Beijing, China). The images were captured using a microscopy (Nikon, Chiyoda-Ku, Tokyo, Japan).

### TOPFlash luciferase assay

Cells were planted in 24-well plates. Then, Wnt/β-catenin TOPFlash plasmids (Addgene, 12,456) as well as mutant FOPFlash plasmids (Addgene, Cambridge, MA, U.S.A.) were co-transfected into the cells, along with *Renilla* TK-luciferase vector (Promega, Haidian, Beijing, China). Then, luciferase detection kits (Promega, Haidian, Beijing, China) was applied to assess the cellular luciferase activity.

### Statistical analysis

Statistical analyses were performed by SPSS (Version 20.0, SPSS Inc., Chicago, U.S.A.). The Student’s *t* test or one-way ANOVA was employed for analyzing the differences between groups. Survival curves were evaluated by Kaplan–Meier method (with log-rank test). A Cox proportional hazards model was used for univariate and multivariate analyses. A value of *P*<0.05 was considered statistically significant.

## Results

### HOXB-AS3 was overexpressed in EOC patients and associated with poor clinical outcome

**Figure 1:**
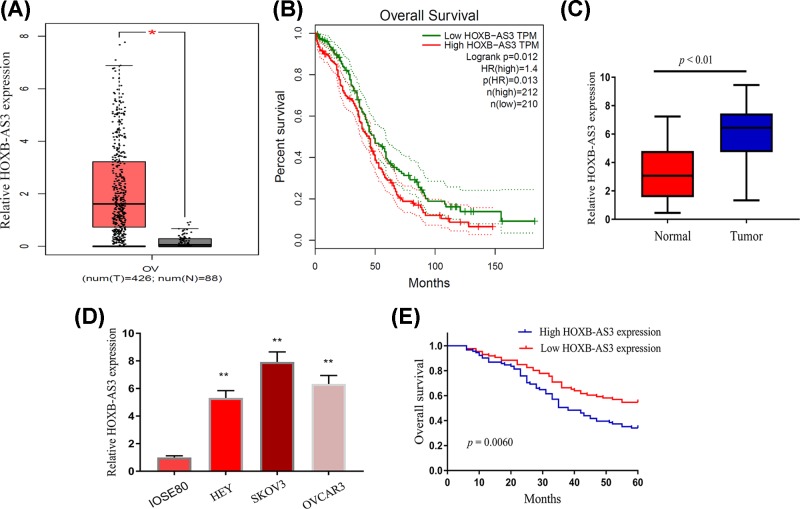
Relative HOXB-AS3 expression and its clinical significance in EOC patients (**A**) The HOXB-AS3 expression levels in EOC tissues compared with normal tissues in TCGA dataset. (**B**) Kaplan–Meier survival assays confirmed that higher HOXB-AS3 correlated with a better overall survival, using TCGA dataset. (**C**) Comparison of HOXB-AS3 expression levels between EOC tissue and adjacent normal tissue by RT-PCR using *U6* as the reference gene. (**D**) The expression levels of HOXB-AS3 in three EOC cell lines (HEY, SKOV3 and OVCAR3) and human ovarian cell line (IOSE80) were detected by qRT-PCR. (**E**) Kaplan–Meier curves for survival time in patients with EOC based on the HOXB-AS3 levels. *P<0.05, **P<0.01. Abbreviation: qRT-PCR, quantitative real-time polymerase chain reaction.

To identify possible lncRNAs that may be involved in EOC and development, we first analyzed lncRNAs expressing profiles by an online tool ‘GEPIA’ which collects clinical data from TCGA database [20]. As shown in Figure 1A, we found that the levels of HOXB-AS3 in EOC tissues was higher than that of the normal ovary samples. Clinical survival assay indicated that higher HOXB-AS3 expression levels were associated with a shorter overall survival (*P*=0.0012, Figure 1B). Thereafter, we performed qPCR to detect EOC tissues and matched normal tissues from patients of our hospital, finding that HOXB-AS3 levels were elevated in EOC tissues compared with matched non-tumor tissues (*P*<0.01, Figure 1C). Besides, the observation also showed that HOXB-AS3 was significantly up-regulated in EOC cell lines (Figure 1D). These results indicated that HOXB-AS3 expression was up-regulated in EOC.

In order to explore the clinical significance of HOXB-AS3 in EOC patients, the median expression level of HOXB-AS3 was used as a cut-off point to divide all 178 patients into two groups (High and Low). As shown in Table 2, the results of Chi-square test indicated that high HOXB-AS3 expression was distinctly associated with histological grade (*P*=0.001), FIGO stage (*P*=0.015) and lymph node metastasis (*P*=0.005). However, there were no significant correlations of HOXB-AS3 expression with other clinical features. To further explore the possible prognostic values of HOXB-AS3 in EOC patients, we carried out Kaplan–Meier analyses, finding that EOC patients with higher expression of HOXB-AS3 had shorter overall survival as compared with the HOXB-AS3-low group (*P*=0.0060, Figure 1E). These results were in line with TCGA data. More importantly, we performed multivariate analysis to explore whether HOXB-AS3 can served as a new biomarker for EOC patients and found that HOXB-AS3 levels was independently associated with overall survival (Table 3).

**Table 2:** Correlation between HOXB-AS3 expression with clinicopathologic features of EOC

Parameters	Category	Number	HOXB-AS3 expression		*P*-value
			High	Low	
Age (years)	≤50	84	40	44	0.377
	>50	94	51	43	
Histological type	Serous	105	55	50	0.687
	Other	73	36	37	
Tumor size	≤5 cm	107	51	56	0.257
	>5 cm	71	40	31	
Ascites	No	66	36	30	0.483
	Yes	112	55	57	
Histological grade	G1	95	38	57	0.001
	G2–G3	83	53	30	
FIGO stage	I–II	99	43	56	0.015
	III–IV	79	48	31	
Lymph node metastasis	No	108	46	62	0.005
	Yes	70	45	25	

**Table 3:** Multivariate Cox regression analysis for overall survival in EOC patients

Variable	Univariate analysis			Multivariate Cox analysis		
	HR	95% CI	*P*-value	HR	95% CI	*P*-value
Age	1.334	0.732–2.132	0.423	-	-	-
Histological type	1.123	0.533–2.042	0.155	-	-	-
Tumor size	1.652	0.839–2.554	0.177	-	-	-
Ascites	1.368	0.932–2.336	0.114	-	-	-
Histological grade	3.323	1.548–6.238	0.001	3.036	1.237–5.546	0.005
FIGO stage	2.846	1.237–4.782	0.008	2.566	1.047–4.132	0.018
Lymph node metastasis	3.223	1.489–5.656	0.006	2.893	1.246–4.778	0.012
HOXB-AS3 expression	3.563	1.638–6.774	0.001	3.126	1.347–5.832	0.003

### Depression of HOXB-AS3 impaired proliferation and promoted apoptosis of SKOV3 and OVCAR-3 cells

**Figure 2:**
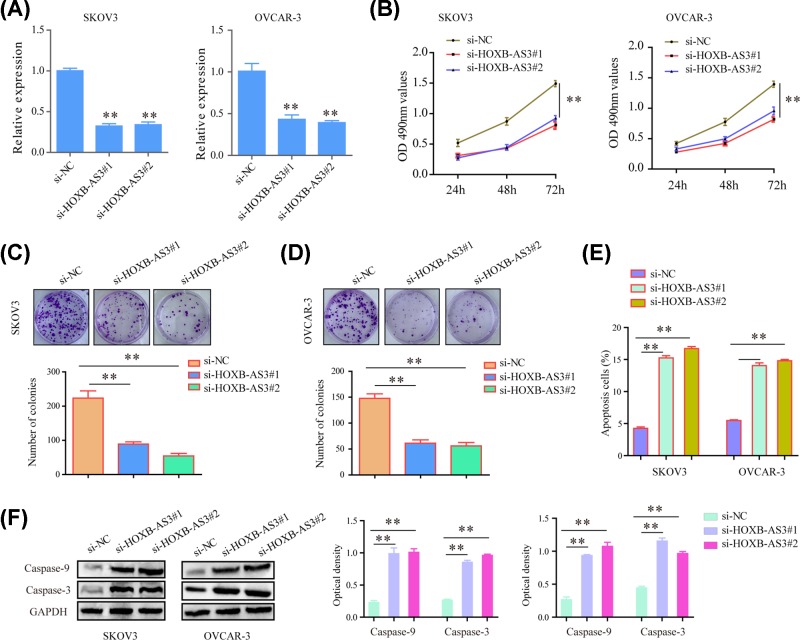
HOXB-AS3 knockdown inhibited the proliferation of EOC cells (**A**) The levels of HOXB-AS3 in SKOV-3 and OVCAR-3 cells using RT-PCR. (**B**) The proliferation rates of SKOV-3 and OVCAR-3 cells detected by MTT assay. (**C,D**) Colony formation assay was conducted. The quantitative number was counted. (**E**) EOC cells transfected with si-HOXB-AS3 or its negative control by flow cytometric analysis with PI staining. (**F**) The protein levels of apoptotic molecular in SKOV-3 and OVCAR-3 cells determined by Western blot assay. **P<0.01.

As the above data proving that HOXB-AS3 was high expression in EOC tissues, we wonder whether HOXB-AS3 played a role in modulating the proliferation and apoptosis of EOC cells. Thus, SKOV3 and OVCAR-3 cell lines were used to perform HOXB-AS3 knockdown experiments. Quantification real-time PCR assay showed that siRNAs (si‐HOXB-AS3#1 and si‐HOXB-AS3#2) transfection caused significant suppression of HOXB-AS3 expression in both EOC cells (Figure 2A). MTT assays demonstrated that the average optical density values at 490 nm were markedly lower in HOXB-AS3 siRNA‐transfected cells than that of the control group in both types of cells (Figure 2B). In addition, colony formation assays certified that HOXB-AS3 siRNA‐transfected cells showed notably impaired growth and grown fewer colonies (Figure 2C,D). Furthermore, flow cytometry revealed that the apoptotic cell percentages were significantly increased in EOC cells transfected with HOXB-AS3 siRNAs (Figure 2E). Besides, the results further suggested that knockdown of HOXB-AS3 led to increased protein levels of caspase 3/9 (Figure 2F). Overall, these data indicated that HOXB-AS3 promoted proliferation and suppressed apoptosis of EOC cells.

### Silencing HOXB-AS3 inhibited EOC cell migration and invasion

**Figure 3:**
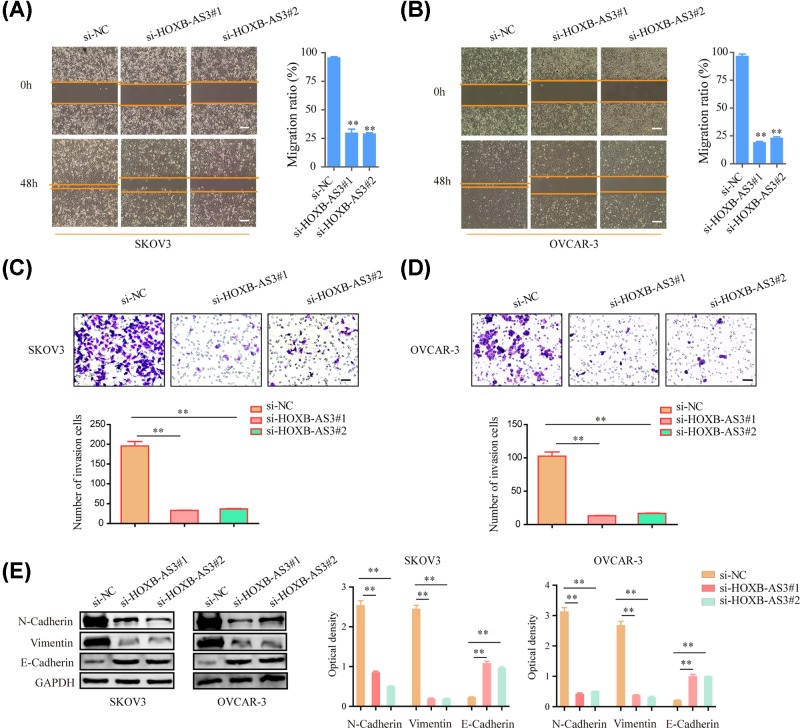
HOXB-AS3 knockdown suppressed OVCAR-3 and SKOV-3 cells migration and invasion (**A,B**) Representative images of the wound-healing assays of HOXB-AS3 knockdown with HOXB-AS3 siRNAs or NC in SKOV-3 and OVCAR-3 photographed at indicated times. (**C,D**) Transwell invasion assay in HOXB-AS3 siRNAs or NC transfected SKOV-3 and OVCAR-3 cells was shown. (**E**) Western blot assay detected the protein levels of N-cadherin, vimentin and E-cadherin in SKOV-3 and OVCAR-3 cells. **P<0.01.

The effects of HOXB-AS3 silencing on the mobility of EOC cells were also investigated by migration and invasion assays. The result confirmed that knockdown of HOXB-AS3 drastically attenuated cellular migration in both SKOV3 and OVCAR-3 cells (Figure 3A,B). Meanwhile, the results of transwell assays suggested that knockdown of HOXB-AS3 significantly impaired the invasive ability of SKOV3 and OVCAR-3 cells (Figure 3C,D). Moreover, the protein levels of epithelial to mesenchymal markers including E-cadherin, N-cadherin and vimentin in EOC cells transfected with HOXB-AS3 siRNAs were further determined. As presented in Figure 3E, knockdown of HOXB-AS3 markedly repressed the N-cadherin and vimentin levels, while the protein expression of E-cadherin was elevated after treatment. Hence, the data elucidated that HOXB-AS3 increased the ability of the migration and invasion of EOC cells.

### Suppression of HOXB-AS3 impaired the activity of Wnt/β-catenin signaling in EOC cells

**Figure 4:**
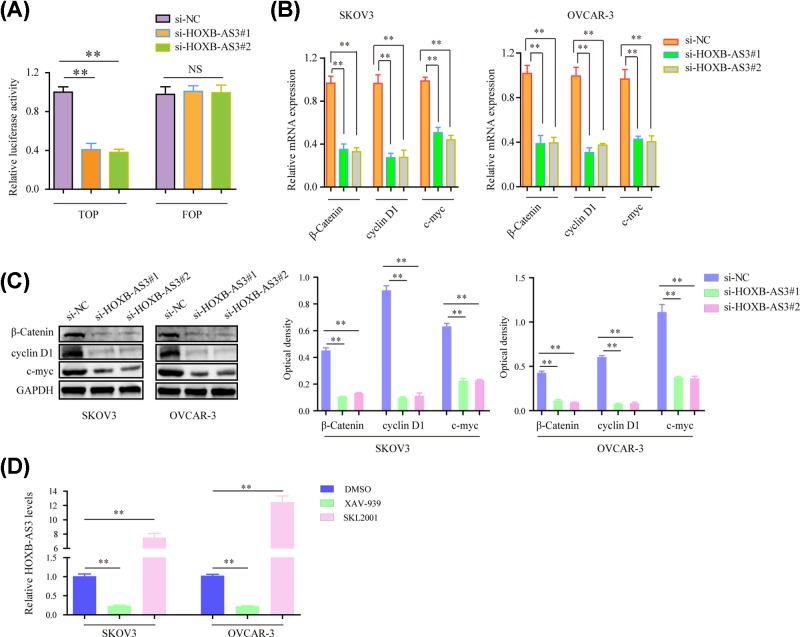
Suppressing HOXB-AS3 affected the Wnt/β-catenin signaling pathway (**A**) TOP-Flash luciferase reporter assays evaluated β-catenin transcription factor/lymphoid enhancer binding factor (TCF/LEF) promoter activity in SKOV-3 cells. (**B**) The mRNA expression levels of β-catenin, cyclin D1 and c-myc were examined by qRT-PCR in SKOV-3 and OVCAR-3 cells. (**C**) Western blot assay for the protein levels of β-catenin, cyclin D1 and c-myc. (**D**) Real-time PCR assays detected HOXB-AS3 expression in EOC cells after treated with Wnt/β-catenin signaling inhibitor (XAV-939) or agonist (SKL2001). **P<0.01.

To explore the underlying molecular mechanisms by which HOXB-AS3 modulated the development and progression of EOC, we next explored the alteration of Wnt/β-catenin signaling in SKOV3 and OVCAR-3 cells. We first carried out TOP/FOP flash reporter assays using SKOV3 cells. The results showed that the luciferase activity of cancer cells transfected with HOXB-AS3 siRNAs was remarkably decreased when compared with the controls, indicating that knockdown of HOXB-AS3 inhibited the activation of the Wnt/β-catenin signaling (Figure 4A). Furthermore, quantitative real-time polymerase chain reaction (qRT-PCR) analysis and Western blot were applied to measure the mRNA and protein expression of Wnt/β-catenin signaling pathway. As the results showed, the mRNA levels of c-myc, β-catenin and cyclin D1 were observed to be significantly decreased in EOC cells treated with HOXB-AS3 siRNAs compared with si-NC-transfected control cells (Figure 4B). Similarly, the dramatically inhibited expressions of β-catenin, cyclin D1 and c-myc at protein levels were also found in HOXB-AS3 knockdown SKOV3 and OVCAR-3 cells (Figure 4C). In addition, qPCR analyses were also utilized to determine the HOXB-AS3 levels in EOC cells after adding Wnt/β-catenin signaling inhibitor (XAV-939) or agonist (SKL2001). The data confirmed that suppressing Wnt/β-catenin signaling significantly reduced HOXB-AS3 levels, while activating Wnt/β-catenin signaling markedly increased the expression of HOXB-AS3 (Figure 4D). Hence, these data demonstrated that knockdown of HOXB-AS3 attenuated EOC development via inhibiting the Wnt/β-catenin signaling activation.

## Discussion

EOC is the most lethal gynecological malignancy in the world and over 75% of EOC patients have already developed metastases when they are first diagnosed; this encourages us to explore novel method targeting early detection and effective treatment [4]. Recently, there have been rising interests in the role of lncRNAs in human diseases, including cancer. In the present study, we first reported the overexpression of HOXB-AS3 in both EOC cell lines and tissues. Clinical data assay revealed that there is a strong association between the high expression of HOXB-AS3 and lymph node metastasis, FIGO stage as well as histological grade, indicating that HOXB-AS3 may act as a positive regulator in EOC progression. Further clinical assay by Kaplan–Meier analyses showed that the survival time of patients was negatively correlated with the expression level of HOXB-AS3. More importantly, based on the result of multivariate analysis, the expression level of HOXB-AS3, as an influencing factor of EOC patients’ survival rate, is independent. Thus, our results, together with the results from ‘GEPIA’ confirmed the significant up-regulation of HOXB-AS3 expression in EOC patients and this up-regulation may serve as a potential independent prognostic value in EOC.

Recently, many lncRNAs have been found to be closely correlated with EOC and function as oncogenes or tumor suppressors [21]. For instance, Cao et al. [22] reported that lncRNA CCAT1, a highly expressed lncRNA in EOC, could promote the invasion, migration and EMT of EOC cells via the modulation of miR-130b and miR-152. Zhang et al. [23] suggested that lncRNA HOTAIR, a well-studied lncRNA in several tumors, was highly expressed in EOC and its silence repressed cells migration by sponging miR-373 in EOC. Yang et al. [24] demonstrated the significant up-regulation of SNHG16 expression in both EOC tissues and cell lines and associated with unsatisfactory survival performance among patients with EOC. *In vitro* experiments indicated that SNHG16 knockdown suppressed EOC cells growth and metastasis. More and more lncRNAs were identified to be link with the development and progression of EOC, making a complete understanding of their biological functions in EOC cells proliferation and metastasis for understanding the potential mechanism of EOC development and metastasis. However, most functional lncRNAs have not been identified.

Recently, a newly identified lncRNA HOXB-AS3 attracted our attention. Previous studies had reported dysregulation of HOXB-AS3 in several cancers, such as acute myeloid leukemia, lung adenocarcinoma and colon cancer [17–19]. However, its clinical significance and biological function, as well as potential molecular mechanism in EOC have not been reported. In the current study, loss-of-function experiments were performed to explore the biological role of HOXB-AS3 in EOC. We found that depression of HOXB-AS3 suppressed proliferation and induced apoptosis of SKOV3 and OVCAR-3 cells by modulating caspase-9 and caspase-3. Furthermore, *in vitro* assay indicated that silencing HOXB-AS3 inhibited EOC cells migration, invasion and EMT. Taken together, our biological experiments confirmed that HOXB-AS3 served as a tumor promoter in EOC because of its effect on EOC cells proliferation, migration and invasion.

Wnt/β-catenin signaling is an evolutionarily conserved pathway and it plays critical roles in both embryonic development and adult homeostasis [25]. It has been confirmed that the Wnt/β-catenin pathway modulated some of the crucial aspects of cellular processes [26]. The perturbations of the Wnt/β-catenin pathway are reported to have association with a number of human diseases, such as cancer, autoimmune diseases and neurological disorders [27–29]. Importantly, Wnt/β-catenin signaling is necessary for the proliferation and metastasis of EOC. Recently, several studies reported that lncRNAs could exert their biological effect by modulating Wnt/β-catenin pathway in various tumors, including EOC [30–32]. Thus, in this study, we wondered whether HOXB-AS3 could regulate the activation of Wnt/β-catenin signaling. Then, we performed Western blot to demonstrate our hypothesis, finding that β-catenin, cyclin D1 and c-myc were dramatically reduced in EOC HOXB-AS3 depletion cell lines. According to the findings, the activation of Wnt/β-catenin signaling in EOC was regulated by HOXB-AS3. Thus, our findings indicated that HOXB-AS3 may exhibit its carcinogenic role by modulating Wnt/β-catenin signaling. Nevertheless, the detailed mechanism remains to be illustrated in further studies.

## Conclusions

The up-regulation of HOXB-AS3 is observed in EOC cell lines and tissues, and its overexpression may be a negative prognostic factor for EOC patients. In addition, HOXB-AS3 can promote tumor cells metastasis by modulating Wnt/β-catenin signaling to inhibit EMT and EOC metastasis. Thus, our study provides basic information to better understand EOC tumor biology and could provide a promise for prognosis or therapy of EOC metastasis.

## Abbreviations

EMT: Epithelial-mesenchymal transition
EOC: epithelial ovarian cancer
FIGO: Federation of Gynecology and Obstetrics
GAPDH: Glyceraldehyde-3-phospgate dehydrogenase
GEPIA: Gene Expression Profiling Interactive Analysis
HOTAIR: HOX transcript antisense RNA
HOXB: Homeobox B cluster
HOXB-AS3: HOXB cluster antisense RNA 3
lncRNA: Long noncoding RNA
qPCR: Real-time Quantitative PCR Detecting System
TCGA: The cancer genome atlas
